# Performance Comparison between Rapid Sequencing Platforms for Ultra-Low Coverage Sequencing Strategy

**DOI:** 10.1371/journal.pone.0092192

**Published:** 2014-03-20

**Authors:** Shengpei Chen, Sheng Li, Weiwei Xie, Xuchao Li, Chunlei Zhang, Haojun Jiang, Jing Zheng, Xiaoyu Pan, Hancheng Zheng, Jia Sophie Liu, Yongqiang Deng, Fang Chen, Hui Jiang

**Affiliations:** 1 State Key Laboratory of Bioelectronics, School of Biological Science and Medical Engineering, Southeast University, Nanjing, China; 2 BGI-Shenzhen, Shenzhen, China; 3 Shenzhen Municipal Key Laboratory of Birth Defects Screening and Engineering, BGI-Shenzhen, Shenzhen, China; 4 Guangdong Provincial Key Laboratory of Human Diseases Genome, BGI-Shenzhen, Guangdong, China; 5 BGI-Nanjing, Nanjing, China; 6 The Key Technology R&D Program of Jiangsu Province, Nanjing, China; 7 Research Group, Complete Genomics, Mountain View, California, United States of America; 8 Department of Stomatology, The Second People’s Hospital of Shenzhen, Shenzhen, China; Natural History Museum of Denmark, University of Copenhagen, Denmark

## Abstract

Ultra-low coverage sequencing (ULCS) is one of the most promising strategies for sequencing based clinical application. These clinical applications, especially prenatal diagnosis, have a strict requirement of turn-around-time; therefore, the application of ULCS is restricted by current high throughput sequencing platforms. Recently, the emergence of rapid sequencing platforms, such as MiSeq and Ion Proton, brings ULCS strategy into a new era. The comparison of their performance could shed lights on their potential application in large-scale clinic trials. In this study, we performed ULCS (<0.1X coverage) on both MiSeq and Ion Proton platforms for 18 spontaneous abortion fetuses carrying aneuploidy and compared their performance on different levels. Overall basic data and GC bias showed no significant difference between these two platforms. We also found the sex and aneuploidy detection indicated 100% sensitivity and 100% specificity on both platforms. Our study generated essential data from these two rapid sequencing platforms, which provides useful reference for later research and potentially accelerates the clinical applications of ULCS.

## Introduction

Ultra-low coverage sequencing (ULCS) is one of the most promising clinical strategies, such as prenatal diagnosis, spontaneous abortion (SA) reason tracking and etc. Although only 0.1X coverage is sequenced for each sample, the data provided efficient genomic information for copy number variation (CNV) detection. Therefore, ULCS strategy is widely used in clinic trials for large-segment CNV detection, such as invasive [1]/noninvasive [2], [3] prenatal diagnosis, Spontaneous Abortion (SA) reason tracking [4]. Unlike karyotyping analysis, ULCS could be applied without cell culture, which is laborious and prone to failure for specific samples, such as SA tissues [5]. In the clinical practice, most of these sequence-based diagnoses have a strict turn-around-time (TAT) requirement, especially for prenatal diagnosis. However, it takes several days for current high throughput platforms to finish a sequencing run; for example, HiSeq 2000 will take approximately 11 days to generate 600 Gbp [6]. Recently, the emergence of rapid sequencing platform, such as MiSeq and Ion Proton, will bring the clinical application of ULCS into a new era.

Targeting clinical laboratories and diagnostic market, in 2011 Illumina released MiSeq, a lower throughput instrument with shorter TAT. With sequencing-by-synthesis and fluorescently labeled reversible-terminator nucleotides technologies, MiSeq currently generates 10 Gbp paired-end 150 bp reads. Benefit from the improved optical system, MiSeq can finish a sequence run within 27-hours [6]. In the same year, Ion Torrent released their Personal Genome Machine (PGM). With the development of integrated semiconductor device, PGM can generate millions of sequential reads by detecting the proton releasing during the base incorporation without any optical systems. By upgrading the semiconductor device, the newly released Ion Proton can generate up to 10 Gbp of sequencing data (with average length of 100 bp) in 4 hours. Recent studies have already showed their feasibility for ULCS clinical application [4]. However, there is a lack of comparison between these two platforms using the same sample set.

In this study, we performed ULCS (<0.1X) to 18 SA fetal tissue samples on both MiSeq and Ion Proton platforms. Also, we compared their performance on GC bias at different level. Afterwards, we investigated sex distinguish and aneuploidy detection performance of both platforms for clinical application. Our research is the first study to generate ULCS data for both platforms using the same sample set, which provides a useful reference of platform selection in the context of clinical especially prenatal diagnosis.

## Results

### Sample Recruitment and Data Generation

To compare the performance between MiSeq and Ion Proton for ULCS, we selected 18 SA fetal samples from a previous study [4]. These fetuses carried 18 different aneuploidies. Their karyotypes are obtained by comparative genomic hybridization and confirmed by FISH. DNA extraction were performed following standard Phenol-Chloroform extraction protocol [4] and quantified with the Quant-iT dsDNA HS Assay Kit (Invitrogen). Considering the amount of input DNA and the PCR process are the most important bias-related factors [7], we used equal amount of input DNA (50 ng) and same numbers of PCR cycles (10 cycles) for library preparation before sequencing on both platforms to minimize the influence to the GC bias comparison (Materials and Methods).

On MiSeq platform, we performed 150 bp paired-end sequencing following the standard manufacture protocol [4]. After a 27-hours sequence run, we generated 4.58 million 150 bp paired-end reads in total for this study, 38.15 Mbp sequence base in average for each sample (Table 1 & Table S1A). 86.35% of these reads could be mapped to the reference genome (GRCh37, UCSC release hg19). After removal of the PCR duplications, ∼214 K paired reads for each sample were obtained, resulting 84.24% effective (unique non-duplicated) reads for the following analysis. On Ion Proton platform, we generated 39.33 million single end reads with a median length of 111 bp. The read length distribution (Figure S1) showed a peak around 127 bp. 87.10% of these single end reads could be mapped to the human genome uniquely. Finally, we obtained 1.70 million unique non-duplicated mapped reads per sample on Ion Proton, which is 7.97 fold more than the data yield on MiSeq.

**Table 1:** Sequencing data statistics.

	MiSeq				Ion Proton			
	Raw		90 K**		Raw		90 K	
	AVERAGE	S.D.*	AVERAGE	S.D.	AVERAGE	S.D.	AVERAGE	S.D.
Reads Number	254,360	36,617	180,000	–	2,184,945	611,149	90,000	–
Unique Map Reads	219,624	32,237	155,513	4,128	1,901,086	526,637	78,400	1,342
Unique Map Rate(%)	86.35	2.27	86.40	2.29	87.10	1.49	87.11	1.49
Duplication Rate(%)	0.08	0.01	0.07	0.01	10.10	0.64	0.62	0.19
Unique non-duplication Reads	214,288	31,740	151,731	4,241	1,707,684	467,757	77,916	1,336
Unique non-duplication Rate(%)	84.24	2.33	84.30	2.36	78.30	1.46	86.57	1.49

*S.D. represents for Standard Deviation.

**90****K paired end reads.

Therefore, to conduct a reasonable comparison, we randomly drew 90****K raw reads from each sample and generated basic statistics of those reads on both platforms (Table 1 & Table S1B). In this equivalent data set, the average duplication rate of Ion Proton data was 0.62%, which was 0.07% on MiSeq. But benefited from lower sequence error rate, it show a higher mapped rate on Ion Proton platform; therefore, Ion Proton left 2.27% more unique non-duplicated reads than MiSeq for further analysis.

### Characteristics of Sequence Bias

**Figure 1:**
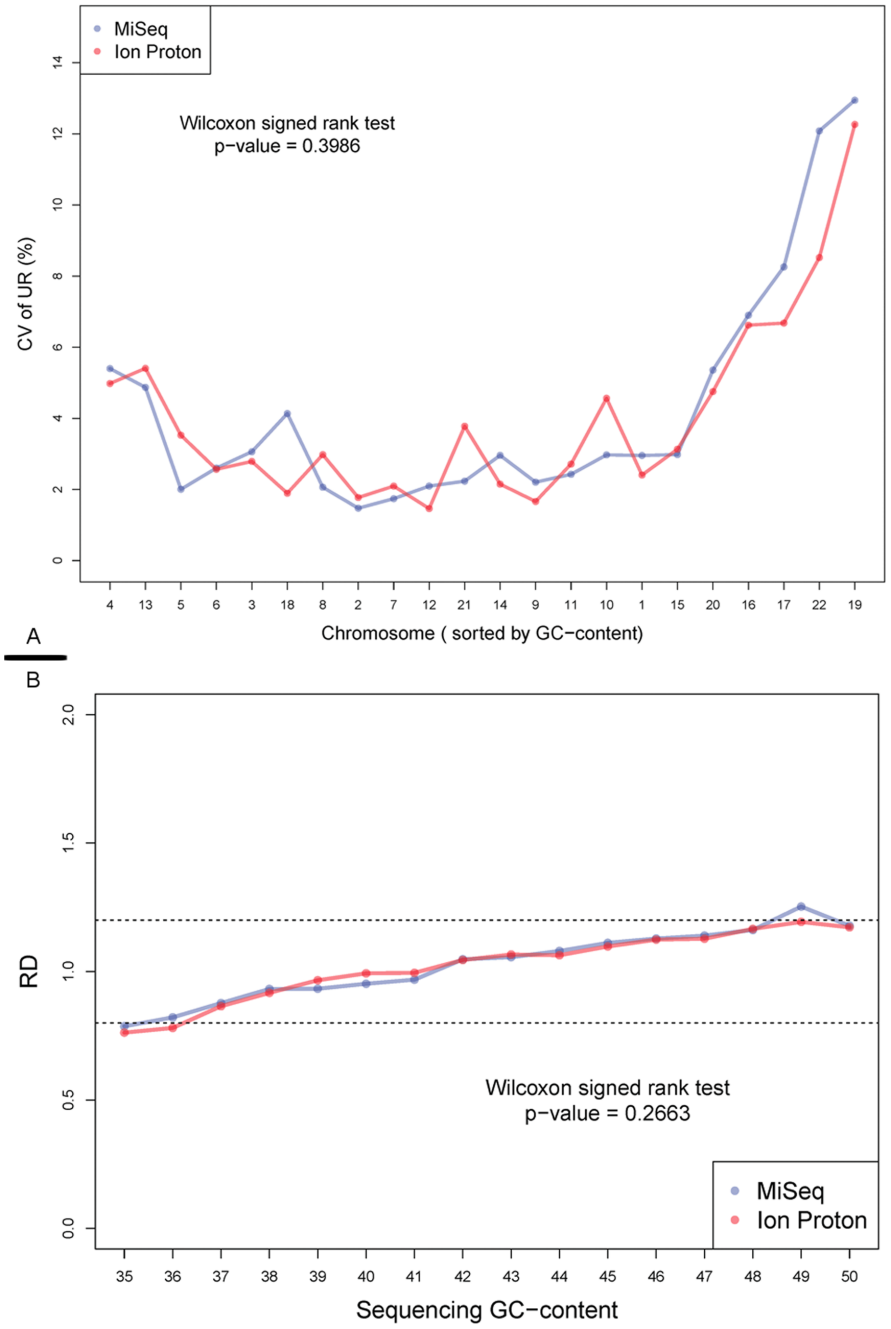
Characteristics of sequencing GC bias. A, Coefficient of Variation of sequencing reads from two platforms on each chromosome. This correlation between CVs of UR% from 18 samples (y-axis) and GC-content of each chromosome (x-axis, sorted by GC content) are shown in this figure. The color-code lines represented data generated on MiSeq (blue) and Ion Proton (red) platform, respectively. B, Average RD in each GC content category. The blue line (MiSeq) and red line (Ion Proton) shows the relative depth (RD, y-axis) at each sequencing GC-content 1 Mbp windows (x-axis). The dash lines represent the RD from 0.80 to 1.20.

**Figure 2:**
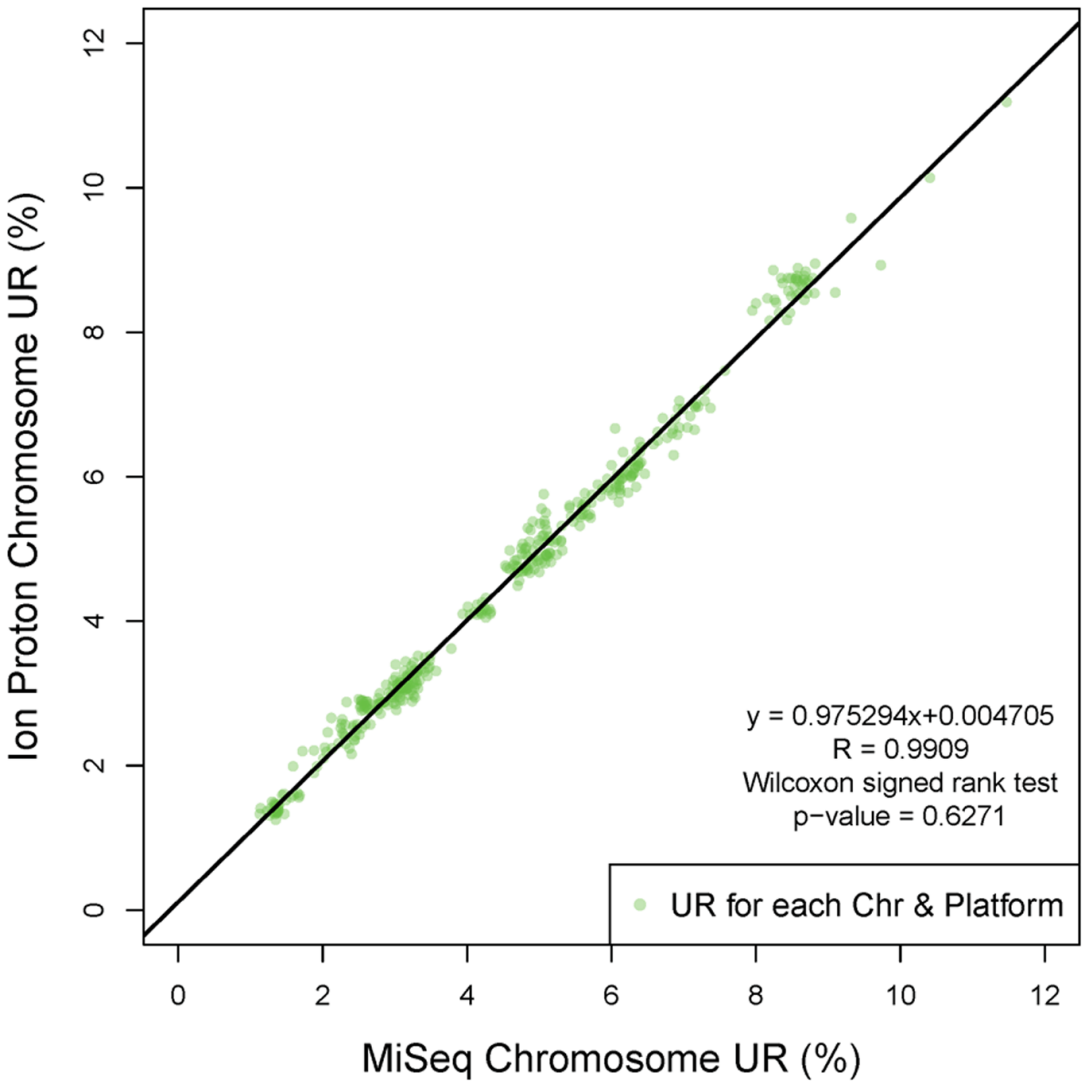
The linear relation between UR% from two platforms. The green scatters show the chromosome UR% calculated using data generated on Miseq (x-axis) and Ion Proton (y-axis) platform, respectively. The linear regression (black line) indicated a good consistency between these two platforms, with a slope of 0.9753, inception of 0.0047 and R^2^ of 0.9909.

Coverage evenness is one of the main factors affects CNV detection, and CNV detection is the most important application of ULCS strategy. Therefore, it’s meaningful to evaluate the coverage evenness in different level. Firstly, we investigated the coefficient of variation (CV) of unique reads percentage (UR%) on each autosome from the 90****K reads data set (Materials and Methods). This CV, which is also employed in a previous study [8], could effectively describe the relative variance of chromosomal UR%, and overall GC bias. The CVs on both MiSeq and Ion Proton were lower than 15% on each chromosome (Figure 1A). The smaller CVs indicated better repeatability for application based from large-scale ULCS, such as aneuploidy screening [8]. The Wilcoxon signed rank test (p-value = 0.3986) indicated there were no significant difference of the autosomal CVs between MiSeq and Ion Proton. Also, we found autosomes with lower (such as chr13) and higher (such as chr19) GC content showed larger CV on both platforms, which is consistent to a previous study [8]. In the consistency analysis, the chromosomal UR% showed high linearity (R^2^ = 0.9909) between MiSeq and Ion Proton (Figure 2) with slope and intercept of 0.9753 and 0.0047 respectively. These numbers implied that these two platforms would display no significant difference on aneuploidy detection.

Considering ULCS would be applied for micro-deletion/duplication syndromes detection, we evaluated the relationship between coverage evenness across GC content at local 1 Mbp window level. The relative depth (RD) of each 1 Mbp observation window was calculated (Materials and Methods).We found most of average RDs for each GC content category fell within the range between 0.8 and 1.20 on both platforms (Figure 1B & Figure S2A/B & Figure S3), which indicates good genome evenness across GC content on both platforms. Besides, we discovered that these two platforms showed similar RD distribution across different GC levels (Wilcoxon signed rank test, p-value = 0.2663). These curves showed reduced RD in GC-poor (GC<38%) regions but increased RD in GC-enrich regions. These analyses implied that current GC bias correction strategies [3] could be employed on these two platforms to detect micro-deletion/duplication syndromes.

### Aneuploidy Detection Power using ULCS

**Figure 3:**
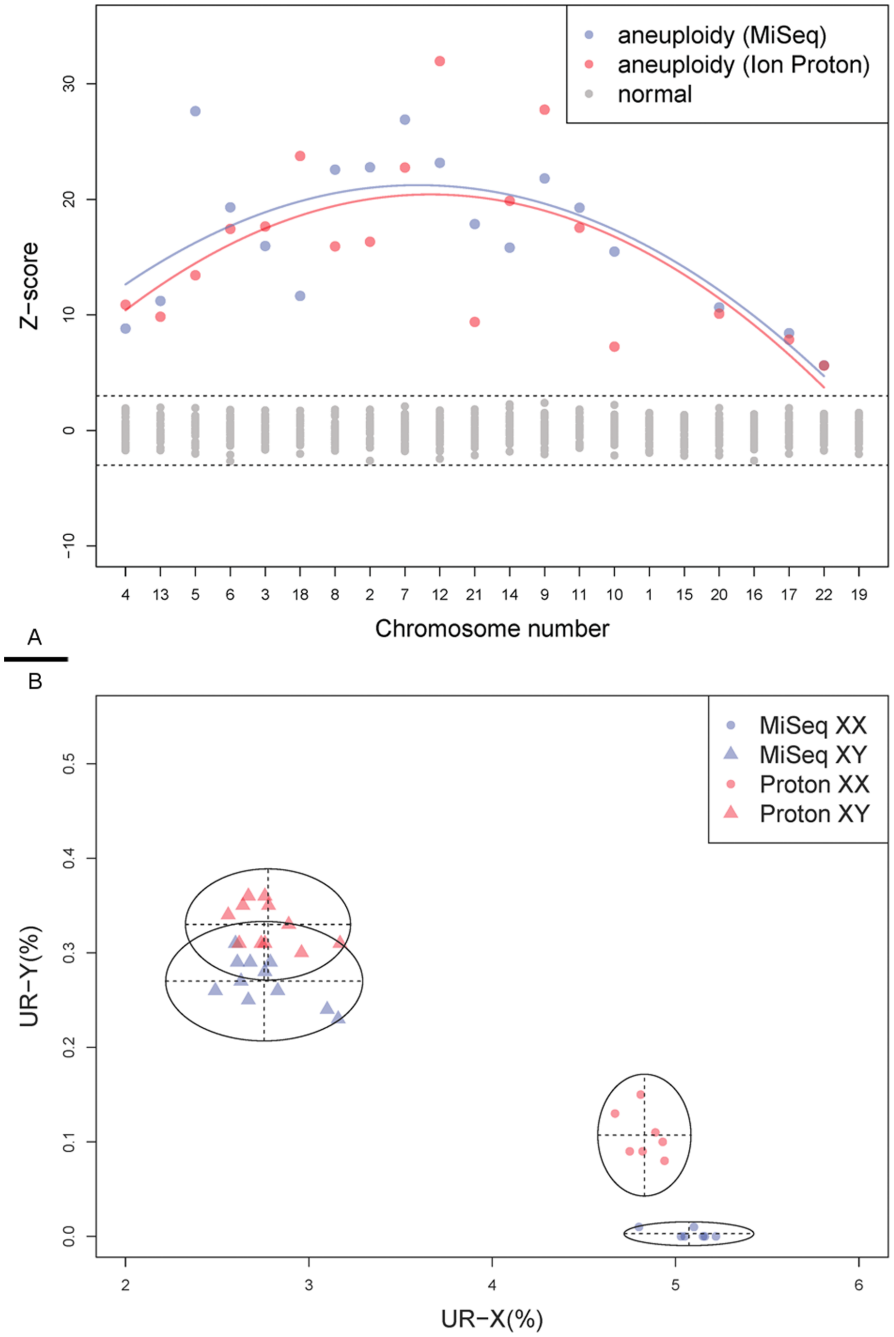
Performance of aneuploidy detection and Sex estimation using 90 K reads from two different platforms. A, Aneuploidy detection result. The z-scores (y-axis) of all 22 autosomes (x-axis, sorted by GC content) for the 18 SA samples are shown in this figure. The cut-off value was set as ±3 (dash line). Aneuploidies signals detected by these two platforms were color-coded (red for MiSeq and blue for Ion Proton) respectively, and negative signals were labeled in grey. The curve was fitted between the z-scores and chromosomal GC content. B, Sex detection with UR-X% and UR-Y%. Using UR-X% (x-axis) and UR-Y% (y-axis), these 18 samples were grouped into two clusters by Gaussian finite mixture model fitted by EM algorithm. These color-coded (red for MiSeq and blue for Ion Proton) scatters with different shapes were label according to the fetal genders (circle for XX and triangle for XY). Each cluster is grouped according to sex detection.

As mentioned, aneuploidies detection is one of the clinical applications of ULCS. To detect aneuploidies among of 18 samples, we employed a Z-score method as described in a previous study [4]. Z-scores enable us to compare the UR% among different chromosomes in a standardized manner; even the chromosomal size and mappability are different. For example, using Z-score method, we can compare UR-chr1 (mean 8.43/8.55 and SD 0.25/0.21) with UR-chr22 (mean 1.43/1.46 and SD 0.17/0.12) effectively, and detect aneuploidies with the same cutoff. Using the equivalent data set, all of the aneuploidies are accurately detected (Figure 3A and Table 2), which indicated 100% sensitivity and 100% specificity for both platforms. This result implied a comparable detection power of these two platforms, presenting their feasibility for clinical applications. However, we realized that it is an incomplete conclusion based on only 18 positive samples; indeed, a larger sample size would be more statistically meaningful.

**Table 2:** Aneuploidy Detection results using UCLS.

Sample	MiSeq		Ion Proton		Standard Karyotype
	Detection	Consistency*	Detection	Consistency*	
SAA01	47,XX +2	Yes	47,XX +2	Yes	47,XX +2
SAA02	47,XY +3	Yes	47,XY +3	Yes	47,XY +3
SAA03	47,XY +4	Yes	47,XY +4	Yes	47,XY +4
SAA04	47,XX +5	Yes	47,XX +5	Yes	47,XX +5
SAA05	47,XX +6	Yes	47,XX +6	Yes	47,XX +6
SAA06	47,XY +7	Yes	47,XY +7	Yes	47,XY +7
SAA07	47,XY +8	Yes	47,XY +8	Yes	47,XY +8
SAA08	47,XX +9	Yes	47,XX +9	Yes	47,XX +9
SAA09	47,XY +11	Yes	47,XY +11	Yes	47,XY +11
SAA10	47,XY +10	Yes	47,XY +10	Yes	47,XY +10
SAA11	47,XX +12	Yes	47,XX +12	Yes	47,XX +12
SAA12	47,XX +13	Yes	47,XX +13	Yes	47,XX +13
SAB01	47,XY +14	Yes	47,XY +14	Yes	47,XY +14
SAB02	47,XX +17	Yes	47,XX +17	Yes	47,XX +17
SAB03	47,XY +18	Yes	47,XY +18	Yes	47,XY +18
SAB04	47,XY +20	Yes	47,XY +20	Yes	47,XY +20
SAB05	47,XY +21	Yes	47,XY +21	Yes	47,XY +21
SAB06	47,XY +22	Yes	47,XY +22	Yes	47,XY +22

*Consistency: We obtain the consistency by comparing the detection result with the standard karyotype.

Interestingly, in the aneuploidy detection, we also found that chromosomal GC content was one of the main reasons affecting the significance of our detection. The curve in Figure 3A indicated trisomy of chromosomes with lower or higher GC content trend to have smaller Z-score than those with average GC content. For instance, the Z-score of chr7 (GC = 40.75%) for SAA06 (47 XY, +7) is 26.90 for MiSeq and 22.75 for Ion Proton, while chr22 (GC = 47.99%) for SAB06 (47 XY, +22) have smaller Z-score of 5.63 and 5.61 for MiSeq and Ion Proton respectively. This phenomenon consistent with the chromosomal CV evaluation.

### Sex Chromosome

Theoretically, the fetal sex can be detected by the UR-Y% easily. However, unexpected aligned read from other genomic region, mostly induced by sequencing error or miss alignment, might lead to unexpected high UR-Y% and false determination of sex. Using the sequence reads from female fetus, we found the unexpected aligned reads mainly enriched in two region on chrY (chrY: 13,104,553–13,748,577 and chrY: 58,819,361–59,373,566) (Figure S4). To achieve higher accuracy, we removed these two regions from the calculation of UR-Y%. Afterwards, we employed a Gaussian finite mixture model fitted by E–M algorithm for sex distinguish (Materials and Methods). In this analysis, these 18 samples were successfully classified into two clusters (Figure 3B). Both platforms showed 100% accuracy for the sex. However, there were still some slight differences between these two platforms. In the female clusters, UR-X% and UR-Y% of MiSeq data (SD = 0.1371 and 0.0049 respectively) showed more narrow and centralized distribution than those of Ion Proton (SD = 0.0985 and 0.0250 respectively), while in the male cluster, UR-X% and UR-Y% of MiSeq data (SD = 0.2082 and 0.0245 respectively) was also more narrow and centralized than the Ion Proton data (SD = 0.1745 and 0.0228 respectively). This phenomenon may relate to the sequence reads length difference between these two platforms. In a previous study, we found the longer sequence reads will improve the accuracy of sex distinguish [4].

## Discussion

In this study, we sequenced 18 SA fetuses on both MiSeq and Ion Proton platforms. By comparing the basic statistics on the equivalent read data set, we found these two platforms showed similar performance. In chromosomal CV analysis, both MiSeq and Ion Proton showed CV lower than 15% on all chromosomes, which was good for following aneuploidy detection. Based on the equivalent data set, we accurately detected the aneuploidies and sex with 100% sensitivity and specificity at high confidence. Moreover, the consistency analysis indicated high comparability between these two platforms.

In the bias analysis, the curve between RD and GC content revealed GC bias on both MiSeq and Ion Proton platforms. Currently, researches attributed this bias to the PCR process during library preparation, assuming more PCR cycles can potentially lead to significant GC bias [7]. Therefore, in this study, same PCR cycles were recruited in the library preparation for both MiSeq and Ion Proton. As expected, both platforms showed similar GC bias, which is consistent with former research and indirectly supports that PCR cycles number is the main factor inducing GC bias. It is conceivable that the surface chemistry processes before sequencing, such as bridge PCR for MiSeq and emulsion PCR for Ion Proton, and the sequencing methods, in the case of ULCS, had contributed less significant GC bias than the PCR step during library preparation. Therefore, methods to reduce the PCR bias during library preparation could potentially improve platform performance for the clinical application of ULCS strategy.

In clinical practice, throughput, cost and TAT are the critical factors restricted their application. Comparison of these two platforms is shown (Table 3). Currently, in each paired-end 150 bp run, Illumina MiSeq needs 24–27 hours to generate up to ∼17 million paired reads, while Ion Proton can generate up to ∼80 million long reads using 3–4 hours with PI chips and 200 bp kit. The shorter TAT of Ion Proton, which enables us to finish the detection within 1∼2 working days, may bring benefits to applications with strict TAT requirement such as prenatal diagnosis. However, the higher throughput will bring both advantage and disadvantages to Ion Proton platform. On one hand, higher throughput will enable us to generate more data for each sample. On the other hand, we need to improve the library preparation for higher coverage sequencing. For example, in raw data for these 18 samples, the duplication rate of Ion Proton platform was as high as 10.10% in average, which decreased the percentage of effective reads. Several optimizations on input DNA amount, enzyme and number of PCR cycles need to be addressed in the future. In the cost aspect, for each gigabyte sequence data, MiSeq and Ion Proton need $150 and $100 respectively (Table 3). Assuming that we need 1 million raw reads for ULCS strategy, MiSeq and Ion Proton will cost $46–53 and $14–15 for each patient/sample respectively. The lower cost of Ion Proton will show advantage on clinical application. The dramatic decrease of TAT and cost make sequencing technology comparable or even superior to some existing approaches, such as comparative genomic hybridization.

**Table 3:** Main output comparisons of current benchtop sequencing platforms for ULCS strategy.

Platform	Reads length	#Base production	#Reads production	Cost	Run time	#Sample per run^c^	Cost per sample
MiSeq	Paired-end 150 bp	Up to 5.1 Gbp	17M^a^	$750^[12]^	24–27 hours	14–16	$46–53
Ion Proton	∼200 bp	Up to 10 Gbp	80M	$1000^b^	3–4 hours	70–75	$14–15

a paired end reads.

b Only include the PI chip ($699), reagent for template ($166) and sequencing ($135), pricing from Invitrogen US territory website (http://www.invitrogen.com/, accessed 2 August 2013).

c Assuming that we need 1M raw reads per sample, and 10% pooling variance.

To sum up, in this study, we generated efficient and essential raw data for MiSeq and Ion Proton using ULCS for 18 samples carrying rare mutations. Also, our comparisons on data production, GC-bias, sex distinguish, TAT and cost provided important reference for following researches. Our evaluation revealed the feasibility for clinical application of ULCS on MiSeq and Ion Proton platforms. Our study has demonstrated the capability of Ultra-Low Coverage Sequencing strategy in clinical applications, more samples are required to make a more comprehensive conclusion.

## Materials and Methods

### Sample Collection and DNA Extraction

In this study, our spontaneous abortions samples were collected between Jan. 2008 and Jan. 2011 from the Institute of Reproduction and Stem Cell Engineering of Central South University, China, and used in former study [4]. CGH and FISH are recruited to obtain and confirm the fetal karyotype. Informed written consent was obtained from each participant, and this study was approved by the Institutional Review Board of BGI and the Institute of Reproduction and Stem Cell Engineering of Central South University.

### MiSeq: Libraries Preparation, Sequencing and Mapping

In brief, for library preparation, we sheared the gDNA into 150–200 bp fragments, performed end repair, “A”-overhangs, and adapter ligation. For more details, we purified the DNA fragments using the Agencourt AMPure Kit (Beckman) and amplified it through 10-cycles PCR with multiplex primers. Index tags were induced during this process. After purification of the PCR products, we used Agilent Bioanalyzer DNA 1000 Kit (Agilent Technologies) to confirm the size distribution of the libraries, and quantified them by real-time quantitative PCR. We then pooled the libraries in equal molecular amounts and sequenced with 150 paired-end cycles on the Illumina MiSeq following the standard protocol. The data has been uploaded to NCBI SRA database (SRA116521).

After all, sequencing reads were mapped to the human genome reference (GRCh37, UCSC release hg19) using SOAP2 [9], and only unique non-duplicated reads were used for the follow-up aneuploidy analysis.

### Proton: Libraries Preparation, Sequencing and Mapping

In library preparation, we first treated the fragment DNA with T4 DNA polymerase, T4 phosphonucleotide kinase and the Klenow fragment of Escherichia coli DNA polymerase for end repair. Then we ligated the sequence adapter (with 10-bps barcode) to the repaired DNA, and performed nick repair. Afterwards, we amplified the size selected DNA through a 10-cylce PCR. After PCR, we confirmed the library size distribution and quantified the libraries using Agilent Bioanalyzer 2100.

For template preparation procedure and sequencing, we followed the Ion Proton manufacturer’s standard protocol. We pooled the libraries at equal moles, and conducted the template preparation using the Ion PI Template OT2 200 Kit on the Ion OneTouch™ 2 System. According to the user’s guide, we recovered and enriched the Ion Sphere Particles (ISPs), and used the Ion Sphere TM Quality Control assay with the Qubit 2.0 Fluorometer for quality assurance. Afterwards, we loaded the qualified ISPs with template into the Ion PI chips for sequencing. On the Ion Proton System, we used Ion PI Sequencing 200 Kit at 400 flows following the manufacturer’s instruction. Also, the data has been uploaded to NCBI SRA database (SRA116521).

The sequencing reads were mapped to the human reference genome (GRCh37, UCSC release hg19) using Tmap [10]. After removal of PCR duplication, the rest uniquely mapped reads were used for the following analysis.

### Aneuploidy Detection

To detect the aneuploidies, we employed the Z-score method in this study. Firstly, we calculated the unique reads percentage (UR%) for each chromosome (labeled as subscript 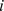, 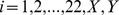) of each sample (labeled as subscript 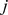, 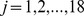). The UR%, termed as 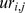, was defined as the ratio between the count of unique reads from chromosome 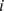 and all autosomes. Secondly, the mean value (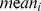) and the standard deviation (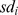) of the UR% of chromosome 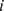 were calculated. For an affected chromosome 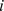, samples unaffected for that chromosome served as the control set. The Z-score was calculated for each chromosome as follows:
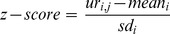



A chromosome with a Z-score≥3 was classified as a trisomy, while the chromosome with a Z-score≤−3 classified as a monosomy. All unaffected chromosomes would present −3<Z-score<3.

### Sex Distinguishing

For sex distinguishing, we recruited mclust (http://www.stat.washington.edu/mclust/), a package of Gaussian finite mixture model fitted by EM algorithm [11] in R. For more details, UR-X% and UR-Y% are calculated and given to mclust (modelNames = “VVI”, G = 2) as two-dimension raw input. After the iteration, our samples would be divided into 2 clusters, meanwhile, parameters such as mean, SD and etc. would be estimated using maximum-likelihood method. The sex of these two clusters would be determined by their average UR-X% and UR-Y%; in brief, the male cluster would show smaller UR-X% but higher UR-Y% comparing with female cluster.

## Supporting Information

Figure S1 **The distribution of Ion Proton sequencing reads length.** The Ion Proton sequencing reads length (x-axis) distribution. Furthermore, this distribution represents a peak-value at 127 bp, also median reads length is 111 bp.
(TIF)

Figure S2 **The distribution between relative depth and sequencing GC-content.** These boxplot show the relationship between sequencing relative depth (RD, y-axis) and their GC-content (x-axis) of each 1 Mbp observation window by MiSeq (Figure A) and Ion Proton (Figure B), respectively.
(TIF)

Figure S3 **Sequencing relative depth of MiSeq and Ion Proton.** Sequencing relative depth for MiSeq (x-axis) and Ion Proton (y-axis) was represented as heat-map. It showed the high level of consistency. The color strength represents the extent of association (from black (strongest), red, yellow, green (weakest)).
(TIF)

Figure S4 **Sequencing reads from female fetus mapped to chrY.** The effective depth (y-axis) calculated by sequence reads from female fetus using 1 Mbp windows are displayed as bar-plot with their mapped position at chrY (x-axis). The dash line shows the position of centromere. The fold line shows the long N region at Y-q12.
(TIF)

Table S1 **A. Sequencing raw data statistics.** B. Sequencing 90 K data statistics.
(DOC)
