# Supplementary figures and images for: Performance Comparison between Rapid Sequencing Platforms for Ultra-Low Coverage Sequencing Strategy
Source: PLoS One. 2014 Mar 20;9(3):e92192. doi: 10.1371/journal.pone.0092192 (PMC3961333; doi:10.1371/journal.pone.0092192)

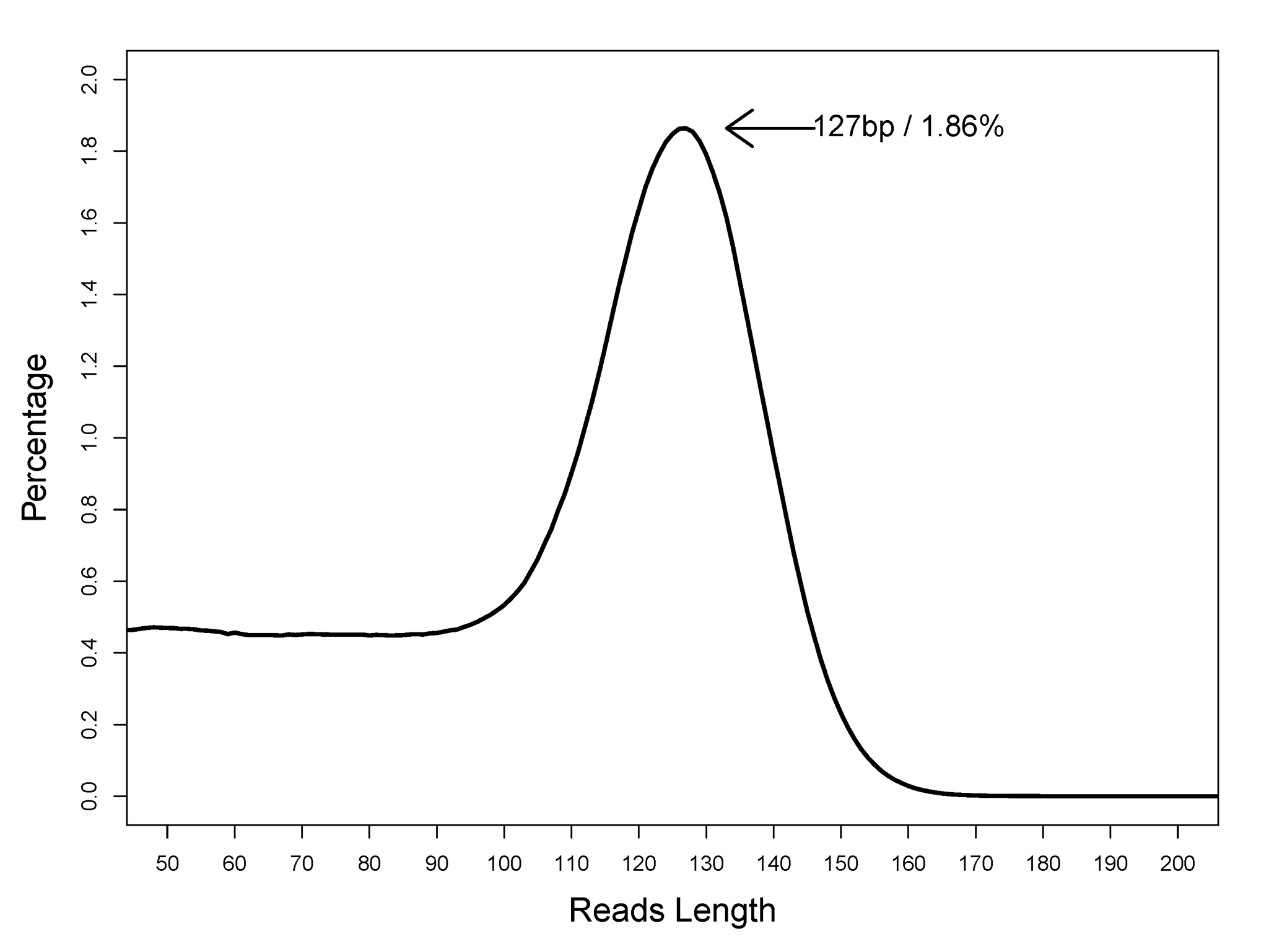

Supplement: Figure S1 — The distribution of Ion Proton sequencing reads length. The Ion Proton sequencing reads length (x-axis) distribution. Furthermore, this distribution represents a peak-value at 127 bp, also median reads length is 111 bp. (TIF) [file pone.0092192.s001.tif]

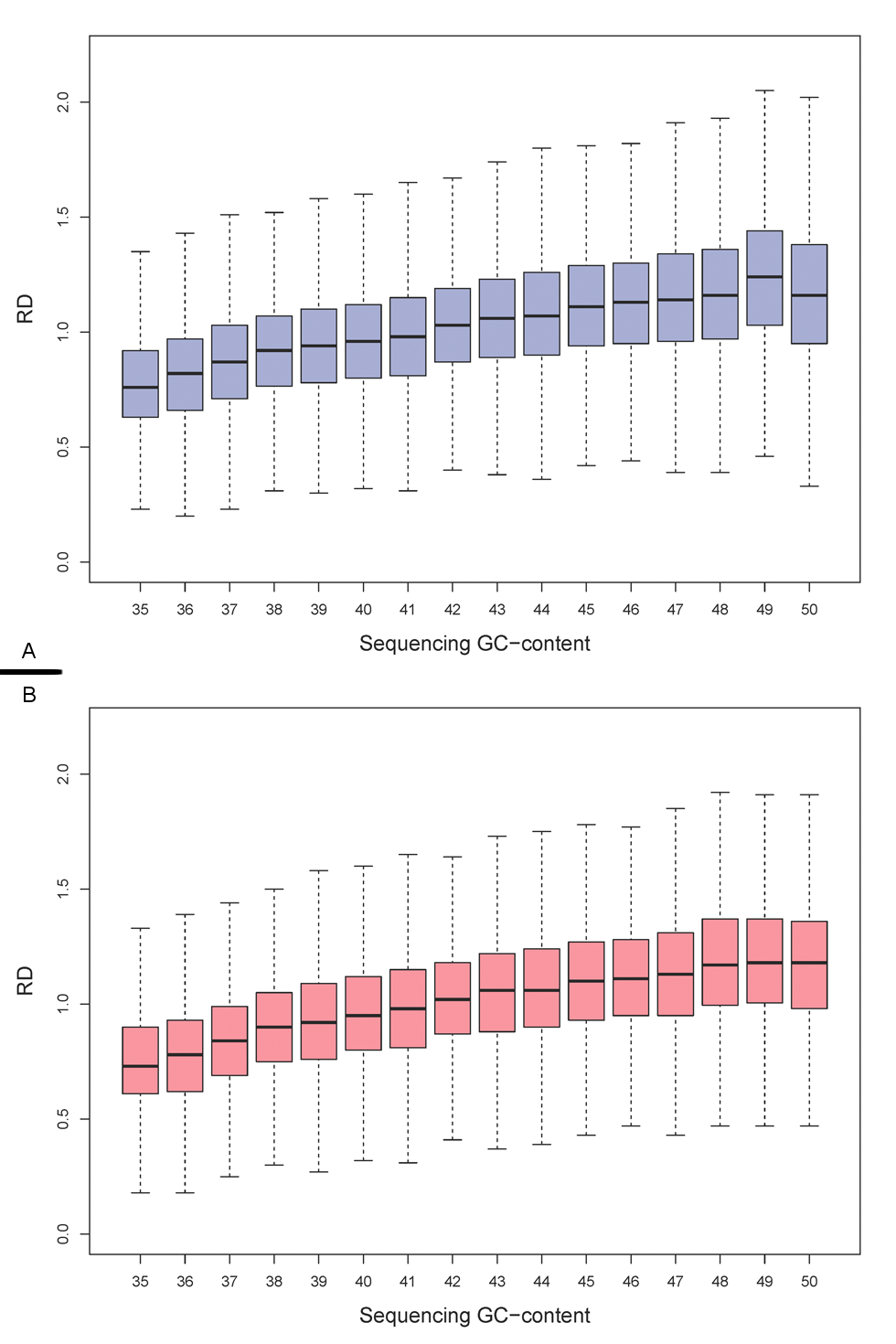

Supplement: Figure S2 — The distribution between relative depth and sequencing GC-content. These boxplot show the relationship between sequencing relative depth (RD, y-axis) and their GC-content (x-axis) of each 1 Mbp observation window by MiSeq (Figure A) and Ion Proton (Figure B), respectively. (TIF) [file pone.0092192.s002.tif]

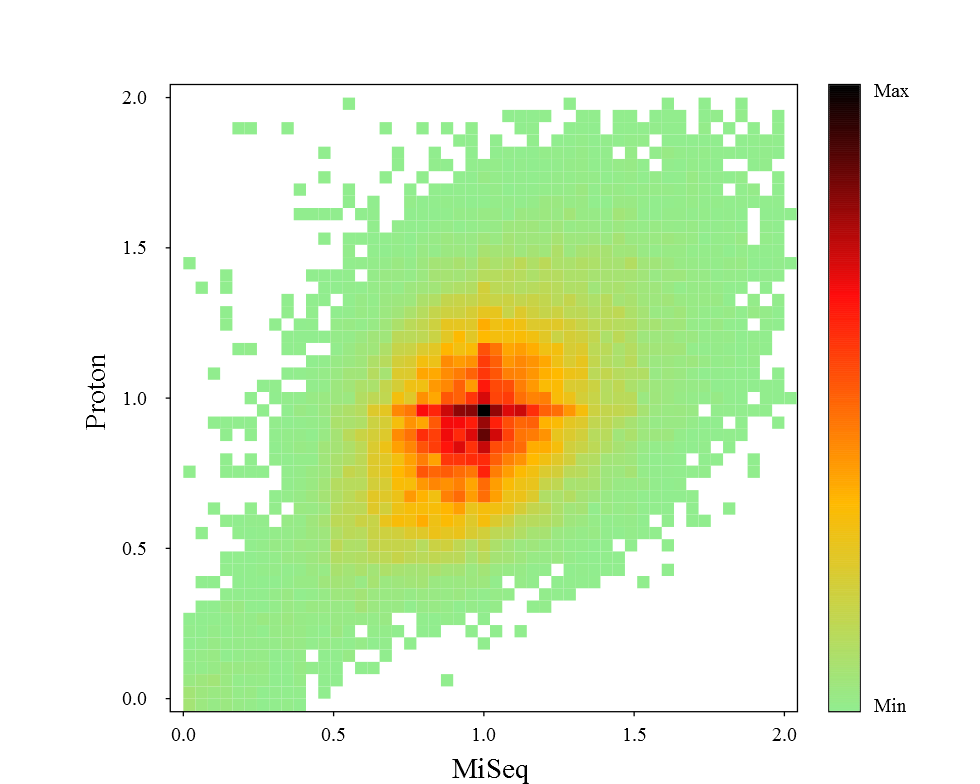

Supplement: Figure S3 — Sequencing relative depth of MiSeq and Ion Proton. Sequencing relative depth for MiSeq (x-axis) and Ion Proton (y-axis) was represented as heat-map. It showed the high level of consistency. The color strength represents the extent of association (from black (strongest), red, yellow, green (weakest)). (TIF) [file pone.0092192.s003.tif]

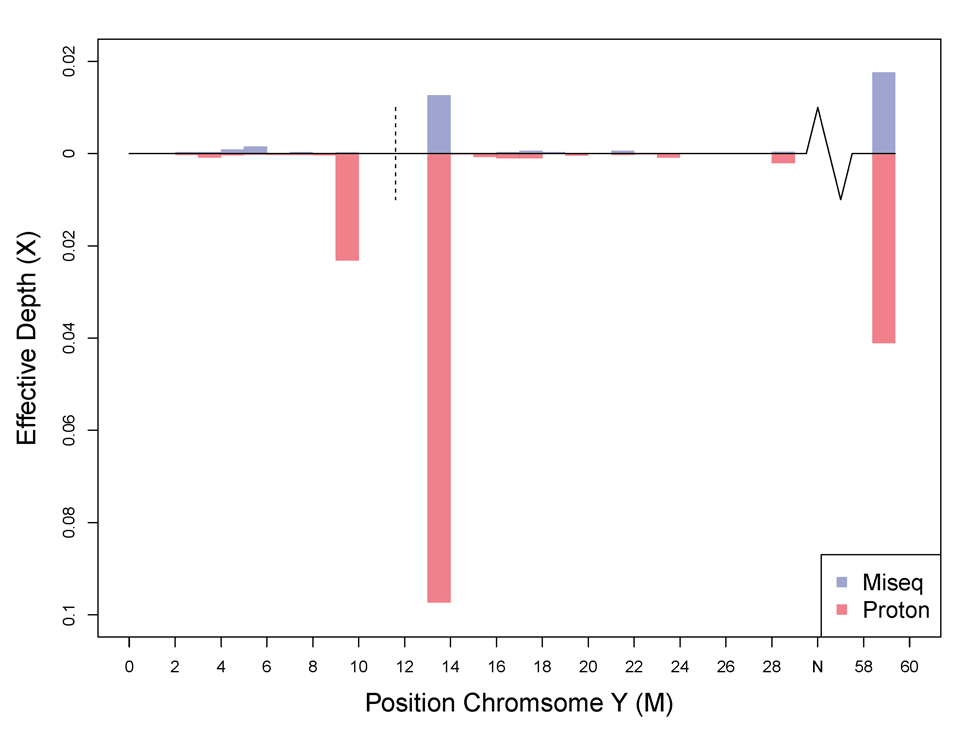

Supplement: Figure S4 — Sequencing reads from female fetus mapped to chrY. The effective depth (y-axis) calculated by sequence reads from female fetus using 1 Mbp windows are displayed as bar-plot with their mapped position at chrY (x-axis). The dash line shows the position of centromere. The fold line shows the long N region at Y-q12. (TIF) [file pone.0092192.s004.tif]
