# Supplementary material for: Performance Comparison between Rapid Sequencing Platforms for Ultra-Low Coverage Sequencing Strategy
Source: PLoS One. 2014 Mar 20;9(3):e92192. doi: 10.1371/journal.pone.0092192 (PMC3961333; doi:10.1371/journal.pone.0092192)
Supplement: Table S1 — A. Sequencing raw data statistics. B. Sequencing 90 K data statistics. (DOC) [file pone.0092192.s005.doc]

*Supporting Information*

**Table S1A. Sequencing raw data statistics.**

|  | MiSeq | | | | | | Ion Proton | | | | | |
| --- | --- | --- | --- | --- | --- | --- | --- | --- | --- | --- | --- | --- |
| Sample | #Reads | #Map Reads | Map Rate (%) | Duplication(%) | #Unique non-duplication Reads | Unique non-duplication Rate (%) | #Reads | #Unique Map Reads | Unique Map Rate (%) | Duplication(%) | #Unique non-duplication Reads | Unique non-duplication Rate (%) |
| SAA01 | 311,146 | 272,326 | 87.52 | 0.09 | 266,892 | 85.78 | 2,846,137 | 2,510,293 | 88.20 | 9.80 | 2,264,284 | 79.56 |
| SAA02 | 277,588 | 240,406 | 86.61 | 0.08 | 235,240 | 84.74 | 2,854,018 | 2,467,013 | 86.44 | 10.12 | 2,217,351 | 77.69 |
| SAA03 | 323,668 | 282,712 | 87.35 | 0.09 | 276,038 | 85.28 | 3,279,231 | 2,830,632 | 86.32 | 10.45 | 2,534,831 | 77.30 |
| SAA04 | 277,756 | 246,272 | 88.66 | 0.10 | 241,142 | 86.82 | 2,083,456 | 1,855,526 | 89.06 | 10.47 | 1,661,252 | 79.74 |
| SAA05 | 255,156 | 218,722 | 85.72 | 0.06 | 213,858 | 83.81 | 1,610,960 | 1,366,094 | 84.80 | 9.28 | 1,239,320 | 76.93 |
| SAA06 | 200,474 | 171,708 | 85.65 | 0.05 | 166,686 | 83.15 | 1,610,169 | 1,429,347 | 88.77 | 9.61 | 1,291,987 | 80.24 |
| SAA07 | 181,276 | 159,618 | 88.05 | 0.08 | 156,102 | 86.11 | 2,258,913 | 1,962,318 | 86.87 | 9.99 | 1,766,282 | 78.19 |
| SAA08 | 273,728 | 216,866 | 79.23 | 0.10 | 211,926 | 77.42 | 2,310,920 | 1,941,404 | 84.01 | 9.52 | 1,756,582 | 76.01 |
| SAA09 | 208,650 | 178,950 | 85.77 | 0.09 | 174,038 | 83.41 | 2,963,333 | 2,533,650 | 85.50 | 11.79 | 2,234,933 | 75.42 |
| SAA10 | 243,652 | 216,336 | 88.79 | 0.07 | 210,866 | 86.54 | 1,964,215 | 1,726,349 | 87.89 | 9.48 | 1,562,691 | 79.56 |
| SAA11 | 246,330 | 218,900 | 88.86 | 0.08 | 214,566 | 87.11 | 2,642,080 | 2,344,846 | 88.75 | 10.03 | 2,109,658 | 79.85 |
| SAA12 | 248,520 | 220,100 | 88.56 | 0.08 | 215,460 | 86.70 | 2,398,177 | 2,112,794 | 88.10 | 10.00 | 1,901,515 | 79.29 |
| SAB01 | 276,780 | 238,922 | 86.32 | 0.09 | 232,174 | 83.88 | 1,503,197 | 1,307,030 | 86.95 | 9.89 | 1,177,765 | 78.35 |
| SAB02 | 221,706 | 189,744 | 85.58 | 0.08 | 184,910 | 83.40 | 1,206,153 | 1,075,406 | 89.16 | 9.78 | 970,231 | 80.44 |
| SAB03 | 268,396 | 225,840 | 84.14 | 0.09 | 219,520 | 81.79 | 1,262,178 | 1,108,066 | 87.79 | 10.68 | 989,725 | 78.41 |
| SAB04 | 237,404 | 202,938 | 85.48 | 0.07 | 197,186 | 83.06 | 2,582,112 | 2,236,884 | 86.63 | 11.06 | 1,989,485 | 77.05 |
| SAB05 | 279,672 | 242,582 | 86.74 | 0.08 | 236,418 | 84.53 | 2,249,018 | 1,949,224 | 86.67 | 10.45 | 1,745,530 | 77.61 |
| SAB06 | 246,580 | 210,286 | 85.28 | 0.08 | 204,166 | 82.80 | 1,704,751 | 1,462,676 | 85.80 | 9.42 | 1,324,892 | 77.72 |
| Mean | 254,360 | 219,624 | 86.35 | 0.08 | 214,288 | 84.24 | 2,184,945 | 1,901,086 | 87.10 | 10.10 | 1,707,684 | 78.30 |
| S.D.* | 36,617 | 32,237 | 2.27 | 0.01 | 31,740 | 2.33 | 611,149 | 526,637 | 1.49 | 0.64 | 467,757 | 1.46 |

*S.D. means Standard Deviation.

**Table S1B. Sequencing 90K data statistics.**

|  | MiSeq | | | | | | Ion Proton | | | | | |
| --- | --- | --- | --- | --- | --- | --- | --- | --- | --- | --- | --- | --- |
| Sample | #Reads | #Map Reads | Map Rate (%) | Duplication(%) | #Unique non-duplication Reads | Unique non-duplication Rate (%) | #Reads | #Unique Map Reads | Unique Map Rate (%) | Duplication(%) | #Unique non-duplication Reads | Unique non-duplication Rate (%) |
| SAA01 | 180,000 | 157,592 | 87.55 | 0.07 | 154,524 | 85.85 | 90,005 | 79,402 | 88.22 | 0.39 | 79,092 | 87.88 |
| SAA02 | 180,000 | 155,946 | 86.64 | 0.06 | 152,676 | 84.82 | 89,997 | 77,784 | 86.43 | 0.46 | 77,426 | 86.03 |
| SAA03 | 180,000 | 157,322 | 87.40 | 0.08 | 153,586 | 85.33 | 90,005 | 77,656 | 86.28 | 0.43 | 77,322 | 85.91 |
| SAA04 | 180,000 | 159,912 | 88.84 | 0.09 | 156,640 | 87.02 | 89,998 | 80,116 | 89.02 | 0.58 | 79,651 | 88.50 |
| SAA05 | 180,000 | 154,360 | 85.76 | 0.04 | 150,976 | 83.88 | 90,002 | 76,448 | 84.94 | 0.86 | 75,791 | 84.21 |
| SAA06 | 180,000 | 154,324 | 85.74 | 0.05 | 149,854 | 83.25 | 90,006 | 79,808 | 88.67 | 0.65 | 79,289 | 88.09 |
| SAA07 | 180,000 | 158,542 | 88.08 | 0.08 | 155,048 | 86.14 | 89,997 | 78,243 | 86.94 | 0.48 | 77,867 | 86.52 |
| SAA08 | 180,000 | 142,610 | 79.23 | 0.08 | 139,410 | 77.45 | 90,004 | 75,405 | 83.78 | 0.50 | 75,028 | 83.36 |
| SAA09 | 180,000 | 154,462 | 85.81 | 0.09 | 150,258 | 83.48 | 89,999 | 77,057 | 85.62 | 0.58 | 76,610 | 85.12 |
| SAA10 | 180,000 | 159,874 | 88.82 | 0.06 | 155,820 | 86.57 | 89,999 | 79,100 | 87.89 | 0.54 | 78,673 | 87.42 |
| SAA11 | 180,000 | 160,134 | 88.96 | 0.07 | 157,002 | 87.22 | 89,999 | 79,901 | 88.78 | 0.40 | 79,581 | 88.42 |
| SAA12 | 180,000 | 159,500 | 88.61 | 0.08 | 156,116 | 86.73 | 89,998 | 79,306 | 88.12 | 0.47 | 78,933 | 87.71 |
| SAB01 | 180,000 | 155,684 | 86.49 | 0.09 | 151,246 | 84.03 | 89,998 | 78,343 | 87.05 | 0.88 | 77,654 | 86.28 |
| SAB02 | 180,000 | 154,164 | 85.65 | 0.07 | 150,286 | 83.49 | 90,004 | 80,221 | 89.13 | 0.93 | 79,475 | 88.30 |
| SAB03 | 180,000 | 151,216 | 84.01 | 0.08 | 147,014 | 81.67 | 89,998 | 79,180 | 87.98 | 0.99 | 78,396 | 87.11 |
| SAB04 | 180,000 | 153,708 | 85.39 | 0.07 | 149,310 | 82.95 | 90,002 | 78,041 | 86.71 | 0.63 | 77,549 | 86.16 |
| SAB05 | 180,000 | 156,068 | 86.70 | 0.06 | 152,132 | 84.52 | 90,003 | 77,907 | 86.56 | 0.62 | 77,424 | 86.02 |
| SAB06 | 180,000 | 153,810 | 85.45 | 0.07 | 149,264 | 82.92 | 90,000 | 77,274 | 85.86 | 0.72 | 76,718 | 85.24 |
| Mean | - | 155,513 | 86.40 | 0.07 | 151,731 | 84.30 | - | 78,400 | 87.11 | 0.62 | 77,916 | 86.57 |
| S.D.* | - | 4,128 | 2.29 | 0.01 | 4,241 | 2.36 | - | 1,342 | 1.49 | 0.19 | 1,336 | 1.49 |

*S.D. means Standard Deviation.
